# Pathology in Africa: back to basics

**DOI:** 10.1007/s00428-012-1229-8

**Published:** 2012-03-29

**Authors:** Pieter J. Slootweg

**Affiliations:** 1 Department of Pathology, Radboud University Nijmegen Medical Center, P.O. Box 9101, 6500 HB Nijmegen, Netherlands; 2 Department of Pathology, Kilimanjaro Christian Medical Center, Moshi, Tanzania

In this issue, Berezowski et al. describe their experience as volunteers in a pathology laboratory in Malawi. It is not a surprise that the case mix they were confronted with differs greatly from what is seen in the daily practice of the majority of diagnostic pathology laboratories: almost half of the cases consisting of nonneoplastic lesions and within the group of neoplasias are a large proportion of pediatric cases, which would be rather unusual for a pathology laboratory in the northern hemisphere.

Not only the nature of cases is different but also the stage of development and size of the lesions that are submitted for pathological examination. Tourists on safari trips to Africa usually want to see the big five: lion, leopard, rhino, elephant, and buffalo. Getting to see them requires some luck and effort and this wish is not always fulfilled. It does not take much effort for the volunteering pathologist to encounter her or his own version of the big five: very large uterine fibroids, goiters, ovarian tumors, breast cancers, and lesions sometimes weighing several kilograms.

However, it is not only the difference in the type of lesion and their size that makes a stay in an African pathology laboratory such a rewarding experience. Working there in fact means practicing pathology as it was done where most of us reside not that long ago: before immunohistochemistry and molecular pathology entered the scene. It encompasses looking at slides that quite often are slightly or even quite a bit thicker than the 3 or 4 μm we are accustomed to. There is no room for postponing of decisions by ordering time-consuming additional stains: the diagnosis has to be made on a few or even just one single hematoxylin and eosin-stained slide. Diagnostic skill and acumen that tend to languish in an environment where a brown deposit usually is considered to be more decisive for diagnosis than careful morphological analysis are brought to new life when one is left with nothing else than a microscope of moderate quality, a pair of eyes, and experience.

An issue only briefly alluded to by Berezowski et al. but requiring a little bit more reflection is whether in that setting histopathological examination plays any role in patient management. Quite often, patients are treated without prior biopsy or, in case their specimens are submitted for pathological examination, have already left the hospital and returned to their villages somewhere in a remote area before the pathology report has arrived on the clinician's desk. This precludes any guidance of treatment by pathological analysis, either preoperatively on the basis of a biopsy or postoperatively through examination of a resection specimen.

Therefore, if pathology is to play any role in guiding daily patient care under sub-Saharan African conditions and resources, it should provide a cheap and quick diagnosis—requirements that obviously can be met by diagnostic cytology. Any volunteering pathologist should master this area and should preferably also be capable of performing FNAs, as this allows not only rapid diagnosis and valuable clinical correlations but also offers an opportunity for personal contacts with less privileged people whose daily lives are much more difficult than ours. This experience may make us more humble and lenient in handling the small inconveniences we all regularly encounter in either our private or professional lives.

